# Clinical Value of Transcutaneous PCO_2_ in Free Flap Blood Supply

**DOI:** 10.3390/jcm14197112

**Published:** 2025-10-09

**Authors:** Fangfang Liu, Nannan Han, Lei Wang, Jinxiu Dong, Min Ruan, Youguo Ying

**Affiliations:** 1 Department of Emergency, Shanghai Ninth People’s Hospital, Shanghai Jiao Tong University School of Medicine, Shanghai 200011, China; 122098@sh9hospital.org.cn (F.L.); 118098@sh9hospital.org.cn (L.W.); 116156@sh9hospital.org.cn (J.D.); 2 Department of Oral and Maxillofacial-Head and Neck Oncology, Shanghai Ninth People’s Hospital, Shanghai Jiao Tong University School of Medicine, Shanghai 200011, China; 124012@sh9hospital.org.cn

**Keywords:** TcPCO_2_, free flaps, oral tumors

## Abstract

**Background:** Transcutaneous PCO_2_ (TcPCO_2_) effectively represents the partial pressure of carbon dioxide in deep tissues, providing us with more accurate information regarding deep tissue perfusion and oxygen metabolism. Based on this, we aimed to explore the clinical value of TcPCO_2_ in assessing free flap blood supply during oral cancer surgery. **Methods:** A total of 27 patients undergoing oral cancer reconstruction with free flap reconstruction were enrolled. For enrolled patients, continuous monitoring was conducted before, during, and after free flap transplantation surgery. **Results:** A total of 121 measurements were taken, comprising 93 instances in the normal flap group and 28 instances in the insufficient flap group. The TcPCO_2_ levels were significantly higher and transcutaneous PO_2_ (TcPO_2_) levels were lower in the insufficient group (*p* < 0.001). The cutoff values for TcPCO_2_ and TcPO_2_, calculated using the Youden index, were 66 mmHg and 16 mmHg, respectively. TcPCO_2_ exhibits high specificity in monitoring the blood supply of free flaps. The area under the ROC curve (AUC) for TcPCO_2_ in predicting insufficient flap perfusion was calculated to be 0.912. **Conclusions:** TcPCO_2_ demonstrates high specificity in assessing blood supply in free flaps for patients undergoing oral cancer surgery and has diagnostic significance for early identification of insufficient flap.

## 1. Introduction

Oral and maxillofacial malignant tumors are a serious threat to patients’ health and life. Free flap transplantation has been widely used in the repair and reconstruction following oral tumor surgeries. Adequate blood supply is crucial for the survival of the transplanted flap. Early detection of blood flow impairment in the flap and timely intervention are vital for the survival of free flaps post-surgery [1]. Various methods have been reported for monitoring flap viability, including near-infrared spectroscopy [2,3], laser Doppler [4,5], immunofluorescence techniques [6], and thermal imaging vascular detection technologies [7,8]. However, no single ideal monitoring technique has been established for widespread application [9]. Currently, clinical observation remains the gold standard for assessing flap blood supply [10,11], but this method cannot provide real-time monitoring. The results are influenced by the observer’s experience and environmental factors, introducing a degree of subjectivity and often leading to a significant delay between the detection of flap damage and intervention. Therefore, there is a need for a simple, reliable, non-invasive, continuous, and low-cost monitoring method to confirm the blood flow status of flaps [12].

Previous studies have primarily focused on PO_2_, with limited literature addressing the changes in PCO_2_. In recent years, transcutaneous PO_2_ (TcPO_2_) and transcutaneous PCO_2_ (TcPCO_2_) monitoring technologies have been applied in various fields, including ventilation function monitoring in intensive care units, postoperative anesthesia monitoring, diagnosis and treatment of limb vascular diseases, and monitoring of peripheral tissue perfusion in the circulatory system [13,14]. Currently, TcPO_2_ is widely applied in flap monitoring, as it can indirectly reflect the oxygen delivery status of target tissues. However, its diagnostic sensitivity is frequently compromised by external confounding factors, including local skin temperature fluctuations and ambient oxygen concentration variations, which limits its reliability in clinical practice. By contrast, carbon dioxide exhibits a diffusion capacity approximately 20-fold higher than that of oxygen; accordingly, TcPCO_2_ directly mirrors the balance between carbon dioxide production and clearance in deep tissues. Notably, an imbalance in this metabolic equilibrium emerges earlier during hypoperfusion—often preceding detectable alterations in TcPO_2_—endowing TcPCO_2_ with potential advantages in early perfusion defect detection. Previous preclinical animal studies have demonstrated that TcPCO_2_ maintains greater stability under dynamic clinical conditions and can identify subtle perfusion abnormalities that may be missed by TcPO_2_ monitoring. In the context of intraoral flap assessment specifically, conventional clinical observations (e.g., flap color and local temperature) are highly susceptible to interference from oral secretions and physical obstruction caused by soft tissue swelling. Meanwhile, traditional monitoring modalities (such as manual pulsation palpation and ultrasonic Doppler flowmetry) exhibit inherent limitations in deep tissue regions, where their ability to capture accurate perfusion signals is substantially reduced. In contrast, TcPCO_2_ monitoring via non-invasive surface probes enables dynamic, real-time assessment, which effectively mitigates the drawbacks of traditional methods. This unique advantage suggests that TcPCO_2_ may serve as a more robust tool for early identification of blood supply disorders in free flaps utilized for oral cancer reconstruction.

Against this backdrop, the present study was designed to address the aforementioned research gaps with three primary objectives: (1) to systematically evaluate the diagnostic performance of TcPCO_2_ in assessing free flap blood supply following oral cancer reconstructive surgery; (2) to comparatively analyze the specificity and predictive value of TcPCO_2_ versus TcPO_2_ for detecting flap perfusion abnormalities; and (3) to determine the optimal cutoff values for TcPCO_2_ and TcPO_2_, thereby establishing a quantitative reference for early identification of insufficient flap perfusion.

## 2. Materials and Methods

### 2.1. Study Subjects and Data Collection

This is an observational study. Patients diagnosed with oral tumors who received treatment at the Ninth People’s Hospital affiliated with Shanghai Jiao Tong University School of Medicine from April 2024 to December 2024 were included in this study. Inclusion criteria: Adult patients diagnosed with oral tumors requiring tumor resection and free flap transplantation. Exclusion criteria: Patients under 18 years of age, hemodynamically unstable patients, those with a history of chronic obstructive pulmonary disease (COPD), local limb blood supply disorders, and those unable to complete measurements.

A total of 27 patients undergoing oral cancer reconstruction with free flap reconstruction were enrolled. For the enrolled patients, continuous monitoring of blood supply before and after free flap transplantation was conducted using a transcutaneous blood gas analyzer. At present, the clinical observation method remains the gold standard for assessing the blood supply of skin flaps. In clinical practice, a skin flap is determined to have poor blood supply if it exhibits paleness or cyanosis in color, is accompanied by edema, shows a decrease in skin temperature, and has poor blood flow after needle puncture. The condition of flap detachment and clinical judgment of poor blood supply after flap anastomosis were defined as the insufficient flap group, while clinical judgment of good blood supply was defined as the normal flap group.

Demographic data, including age, sex, height, and weight, were collected before the study commenced. The TCM4 transcutaneous oxygen and carbon dioxide monitoring device (Radiometer, Denmark) was used to continuously monitor TcPO_2_ and TcPCO_2_ in the local flap tissue after the surgery began. Prior to testing, the room temperature was set at 25 °C, and the combined oxygen and carbon dioxide detector was calibrated at 43 °C with standard gases. The monitoring site is the local flap tissue, which was cleaned and dried. A fixation ring was applied to prevent air leakage, contact gel was injected, and the electrode was secured. Monitoring results were recorded after the values stabilized. In this study, monitoring conditions were strictly controlled. Firstly, the transcutaneous blood gas analyzer was rigorously calibrated before each experiment, with conditions such as temperature and atmospheric pressure standardized. Secondly, electrodes were uniformly placed at the distal part of the local flap; continuous monitoring was initiated after the start of surgery, where each monitoring session lasted 1 h, and data were recorded once the readings stabilized.

To avoid the impact of observers’ subjective bias on the evaluation results of insufficient flap, this study adopted a single-blind design. Specific implementation details are as follows. Observer Qualifications: The personnel responsible for evaluating insufficient flap were 3 attending physicians with more than 5 years of clinical experience in oral and maxillofacial surgery, all of whom had received specialized training in flap blood supply evaluation (including clinical sign identification, imaging interpretation standards, etc.). Blinding Control: Observers only participated in the insufficient flap evaluation process and were not involved in preoperative patient screening, intraoperative monitoring operations, or postoperative recording and analysis of TcPO_2_/TcPCO_2_ data. All patients’ monitoring data (including TcPO_2_ and TcPCO_2_ values as well as trend graphs) were organized and archived by independent research assistants and were only disclosed to the observers after the entire study data was locked. This ensured that the observers were completely unaware of the TcPO_2_/TcPCO_2_ results during the evaluation process.

This study was approved by the Ethics Committee of the Ninth People’s Hospital affiliated with Shanghai Jiao Tong University School of Medicine (Approval No: SH9H-2023-T452-2) and complies with the requirements of the Declaration of Helsinki. All enrolled patients signed informed consent forms.

### 2.2. Sample Size Calculation

Sample size estimation was performed using PASS 2025 software. The following parameters were set: a reference area under the curve (AUC) of 0.5, an expected AUC of 0.75, α of 0.05, β of 0.2, and a positive to negative event allocation ratio of 1:1. The false positive rate ranged from 0 to 1, data type was continuous, and a two-tailed hypothesis test was employed. It was estimated that 18 cases would be needed. Considering potential non-compliance or loss to follow-up, at least 20 cases were required. Since repeated measurements were required for patients at the preoperative, intraoperative, and postoperative stages, combined with the estimated number of sample cases, a minimum of 100 measurements were needed.

### 2.3. Statistical Analysis

Statistical analyses were performed using SPSS version 21.0 and R software (version 4.0.3). Data were presented as mean ± standard deviation or as counts (rates) according to their distribution. A mixed-effects model (with patients as the random effect) was used for statistical analysis to compare the differences between the insufficient flap/poor blood supply group and the flap normal blood supply group. Receiver operating characteristic (ROC) curves were plotted using R software, and a confusion matrix was employed to calculate sensitivity, specificity, negative predictive value, positive predictive value, and accuracy. The optimal cutoff value was determined based on the maximum Youden Index (sensitivity + specificity − 1), with a significance level set at *p* < 0.05 indicating statistically significant differences.

## 3. Results

### 3.1. Baseline Characteristics of Patients

A total of 27 patients undergoing oral cancer resection with free flap reconstruction were enrolled. A total of 121 measurements were recorded using the transcutaneous blood gas analyzer in this study, with 93 measurements from the normal flap group and 28 from the insufficient flap group. Statistical analysis revealed no significant differences in baseline data between the two groups (Table 1).

### 3.2. Comparison of TcPO_2_ and TcPCO_2_ Between the Two Groups

**Figure 1:**
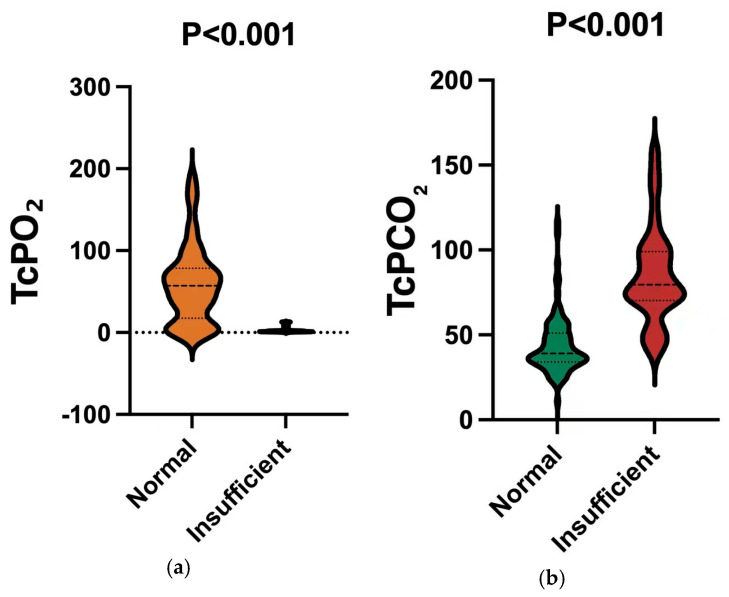
(**a**). Comparison of TcPO_2_ values between different groups. Notes: TcPO_2_ was lower in insufficient (*p* < 0.001). (**b**). Comparison of TcPCO_2_ values between different groups. Notes: TcPCO_2_ was higher in insufficient (*p* < 0.001).

Analysis revealed that in the normal flap group, the TcPCO_2_ was 44 ± 17 mmHg and TcPO_2_ was 59 ± 47 mmHg; in contrast, in the insufficient flap group, the TcPCO_2_ was 85 ± 29 mmHg and the TcPO_2_ was 3 ± 4 mmHg. As shown in the table data, TcPO_2_ exhibits a relatively high degree of variability, and this phenomenon may be closely associated with factors such as TcPO_2_ being susceptible to multiple influences and having poor stability. Although this study has strictly standardized monitoring conditions (e.g., electrode placement and temperature control), TcPO_2_ monitoring inherently relies on skin stratum corneum penetration and local blood flow exchange, making it susceptible to interference from multiple factors. These interfering factors are specifically reflected in three aspects: first, during surgery, the blood flow reperfusion process at the moment of vascular anastomosis can cause a sudden increase or decrease in TcPO_2_ in a short period; second, after surgery, changes in the patient’s body position (such as head and neck torsion that compresses the flap’s blood supply vessels) can also lead to similar short-term drastic fluctuations in TcPO_2_. In addition, in the early postoperative period (6–12 h) for oral cancer patients, the flap is in a reperfusion adaptation phase—during this stage, local blood flow velocity is slow and unstable, resulting in a relatively low average TcPO_2_, which gradually rises as blood vessels dilate. The occurrence of such extreme values directly increases the standard deviation, ultimately leading to high variability in TcPO_2_ values. The comparison showed that patients in the insufficient flap group had significantly lower TcPO_2_ levels and higher TcPCO_2_ levels than those in the normal flap group, with statistical significance noted (*p* < 0.001) (Table 2). A violin plot was generated to provide a more intuitive visual representation of the differences in TcPO_2_ and TcPCO_2_ between the normal flap group and the insufficient flap group (Figure 1a,b).

### 3.3. Analysis of the Value of TcPO_2_, TcPCO_2_, and TcPO_2_ + TcPCO_2_ in Assessing Insufficient Flap

**Figure 2:**
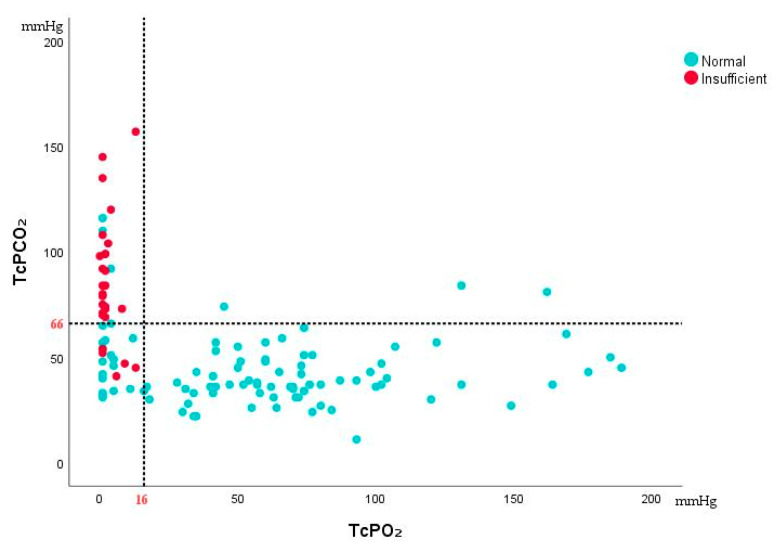
Quadrant scatter plot for TcPO_2_ and TcPCO_2_. Notes: TcPCO_2_ above 66 mmHg and/or TcPO_2_ below 16 mmHg strongly suggest the possibility of insufficient flap.

The sensitivity and specificity were calculated via a confusion matrix, revealing that TcPO_2_ exhibited high sensitivity while TcPCO_2_ showed stronger specificity. Further analysis through calculating the Youden index determined the cutoff values for TcPO_2_ and TcPCO_2_ to be 16 mmHg and 66 mmHg, respectively. (Table 3). In the plotted quadrants of the TcPO_2_ and TcPCO_2_ scatter plot, auxiliary lines at TcPO_2_ = 16 mmHg and TcPCO_2_ = 66 mmHg were included: values in the first quadrant highly suggest insufficient, while those in the fourth quadrant strongly indicate normal flap status (Figure 2). Based on the cutoff values calculated using the Youden index and the four-quadrant scatter plot of TcPO_2_ and TcPCO_2_, we concluded that a TcPO_2_ below 16 mmHg and/or a TcPCO_2_ above 66 mmHg strongly suggest the possibility of insufficient flap. During the continuous monitoring period following vascular recanalization surgery, we observed one patient exhibiting persistently low TcPO_2_ and elevated TcPCO_2_, with the TcPCO_2_ reaching a maximum of 116 mmHg. After clinical assessment by experienced surgeons, the patient’s flap was diagnosed as having poor blood supply; consequently, the decision was made to perform a secondary anastomosis surgery. After active secondary surgery, the skin flap finally recovered well.

**Figure 3:**
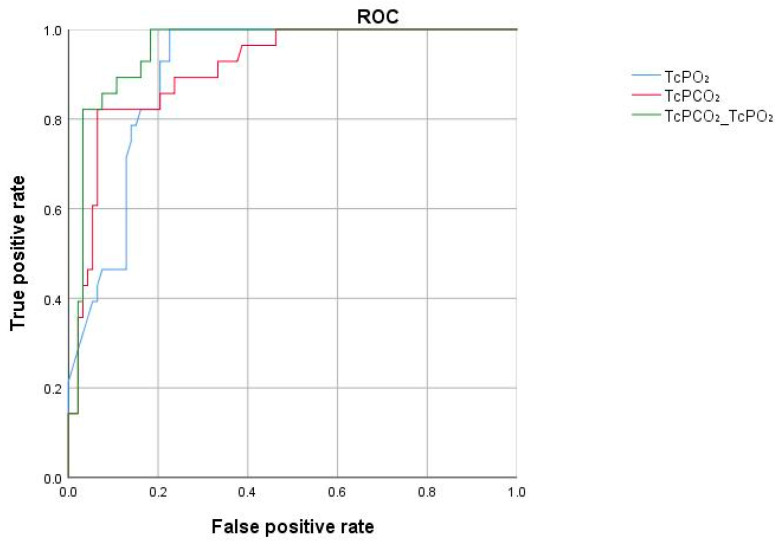
ROC curves for predicting insufficient flap using TcPO_2_, TcPCO_2_, and TcPO_2_ + TcPCO_2_. Notes: The AUC values for TcPCO_2_, TcPO_2_, and TcPO_2_ + TcPCO_2_ in predicting insufficient flap were 0.912, 0.905, and 0.955, respectively.

The ROC curves were plotted for TcPO_2_, TcPCO_2_, and the combined analysis of TcPO_2_ + TcPCO_2_ for predicting insufficient flap, and the areas under the curve (AUC) were calculated and compared. The results showed that the AUC values for TcPCO_2_, TcPO_2_, and TcPO_2_ + TcPCO_2_ in predicting insufficient flap were 0.912, 0.905, and 0.955, respectively. The predictive value of TcPCO_2_ was found to be greater than that of TcPO2, and their combined predictive value surpassed that of either TcPO_2_ or TcPCO_2_ used alone (Figure 3).

## 4. Discussion

With the advancement of microsurgery, free tissue reconstruction has become the primary approach for repairing defects in the oral and maxillofacial regions, with free flap transplantation success rates reaching over 94% [15]. However, various factors can lead to vascular crises resulting in insufficient flap, causing significant suffering and even threatening patients’ lives [16]. Adequate blood supply is crucial for the survival of flaps after transfer. Clinical observation remains the current standard for diagnosing inadequate blood supply or poor venous return after flap transplantation. This approach involves assessing flap color, surface temperature, bleeding upon needle puncture, capillary refill, tissue firmness and swelling, which are cost-effective, simple, and rapid methods for monitoring flap perfusion. However, this method relies heavily on the clinician’s experience and lacks objective measurable support. An ideal flap monitoring technique should possess characteristics such as continuous, precise, non-invasive, recordable, repeatable, and highly sensitive predictive capabilities, yet no such ideal blood supply monitoring technology currently exists.

Previous studies on flap blood supply have primarily focused on oxygen partial pressure, with little attention given to the relationship between carbon dioxide partial pressure and flap blood supply [17]. The transcutaneous blood gas analyzer used in this study incorporates Clark-type PO_2_ and Severinghaus-type PCO_2_ sensors, enabling simultaneous monitoring of TcPO_2_ and TcPCO_2_ [18,19].

The basis of tissue metabolism is aerobic metabolism, and TcPO_2_ serves as the final stage of oxygen diffusion. It is typically the first to be compromised and the last to recover, making it a sensitive quantitative indicator of peripheral perfusion [20,21]. Earlier studies have confirmed that TcPO_2_ can serve as a rapid indicator of vascular impairment. In this study, we found that the TcPO_2_ levels in the insufficient flap group were significantly lower than those in the normal flap group. However, the oxygen diffusion capacity is limited; TcPO_2_ is significantly influenced by epidermal thickness, local gland metabolism, and vascular structure, complicating the achievement of stable values in practical applications [22,23].

Due to its superior diffusion capacity, TcPCO_2_ is significantly less influenced by external factors compared to TcPO_2_. Research on the monitoring of TcPO_2_ and TcPCO_2_ has indicated that TcPCO_2_ values exhibit a narrower range compared to the wide variation in TcPO_2_ values [24]. Previous studies investigating the relationship between TcPCO_2_ and flap blood supply have primarily focused on animal experiments. Abe et al. [25] studied the changes in TcPO_2_ and TcPCO_2_ using rabbits and found that TcPCO_2_ significantly increased during insufficient flap Rochat et al. [26] conducted a study on random flaps in dogs and reported an average TcPCO_2_ of 52 mmHg measured at the base of the flap, while TcPCO_2_ values reached up to 106 mmHg at the flap’s tip. Clinical studies, by contrast, are relatively scarce. The clinical evaluation of TcPCO_2_ monitoring has also been limited to cases of insufficient skin caused by conditions such as necrotizing fasciitis, bullous pyoderma, atherosclerosis, and pressure ulcers. For flaps specifically, previous studies have often focused on changes in transcutaneous carbon dioxide partial pressure across different types of flaps. For instance, Hashimoto et al. [27] monitored transcutaneous carbon dioxide partial pressure in various flaps, including anterolateral thigh flaps, rectus abdominis myocutaneous flaps, scapular flaps, and latissimus dorsi myocutaneous flaps. They confirmed that TcPCO_2_ increases significantly when insufficient flap occurs. However, flap transplantation sites differ in their blood supply requirements. This study focuses on patients with free flaps for oral cancer—an area characterized by complex anatomy and significant fluctuations in blood supply. Compared with previous research, this study fills the evidence gap in the field of complex reconstructive surgery and provides new insights into the individualized and scenario-specific application of TcPCO_2_ monitoring.

TcPCO_2_ is based on the phenomenon that CO_2_ gas easily diffuses into body tissues and the skin, allowing it to be detected via sensors on the skin surface. When the sensors of the transcutaneous blood gas analyzer are heated to a certain temperature, it causes dilation of the skin capillaries, which further increases the permeability of CO_2_ and enhances the delivery of arterial blood to the dermal capillary network beneath the sensor. The CO_2_ gas that diffuses subcutaneously is separated from the skin through a highly permeable membrane (electrode membrane) and dissolves in the electrolyte solution within the electrode, altering its pH. The change in pH is logarithmically proportional to the change in PCO_2_, allowing for the determination of PCO_2_ values [28]. TcPCO_2_ monitoring has been developed over many years and has been widely validated for use in monitoring tissue perfusion in critically ill patients. TcPCO_2_ is fundamentally and physiologically a circulating variable, dependent on systemic and local skin perfusion conditions. During circulatory failure, a “disconnection” occurs between PaCO_2_ and TcPCO_2_, leading to tissue hypercapnia that is unrelated to PaCO_2_. The perfusion status of the flap refers to the local blood supply to the flap tissue after transplantation or reconstructive surgery. The viability of the flap is closely related to its perfusion status; if blood supply is insufficient, the likelihood of insufficient flap increases. Most monitoring techniques for flap blood supply focus on the direct measurement of local hemodynamics, specifically blood flow. However, the pathophysiological basis for vascular crises in transplanted flaps involves a decrease in flap blood flow due to arterial and/or venous obstruction, resulting in insufficient tissue perfusion and a direct or indirect decline in oxygen supply levels, which fails to meet the metabolic needs of flap tissue, leading to reduced aerobic metabolism and increased anaerobic metabolism. TcPCO_2_ is influenced by three main phenomena: (I) the production of CO_2_ by tissues (VCO_2_), (II) the clearance of CO_2_ from tissues via perfusion (washout phenomenon), and (III) the arterial CO_2_ content [18]. Local tissue perfusion can be summarized as the so-called “Tc-a-PCO_2_ gap.” For patients with oral tumors, PaCO_2_ levels generally remain constant, allowing TcPCO_2_ to reflect local tissue perfusion. In preliminary studies, we found that the trend in TcPCO_2_ changes can dynamically reflect the blood supply status of the flap. Before and after free flap transplantation, as perfusion diminishes, TcPCO_2_ gradually increases, reaching a maximum of 135 mmHg, while following vascular anastomosis and blood reperfusion, TcPCO_2_ shows an opposite trend. This study further compared the TcPCO_2_ values between the insufficient flap group and the normal flap group, revealing that TcPCO_2_ was significantly higher in the insufficient group. Based on the cutoff values we concluded that TcPCO_2_ above 66 mmHg strongly suggest the possibility of insufficient flap. In recent years, Nakano et al. [29] conducted a retrospective analysis of patients undergoing tissue reconstruction and found that in 11 cases requiring reoperation, postoperative TcPCO_2_ values exceeded 70 mmHg. The cutoff values we obtained for TcPCO_2_ are consistent with previous studies.

This study has some limitations. This study defines “flap avulsion” as poor flap blood supply, which, while encompassing flap perfusion abnormalities during arteriovenous crises of anastomosed vessels, fails to detect early microcirculatory disorders in flaps caused by ischemia–reperfusion injury or inflammatory factor release. Therefore, subsequent research should further strengthen studies on microcirculatory disorders in free flap tissue to enable earlier identification of free flap perfusion impairment. Additionally, this study may be affected by potential confounding factors: different oral tumors, flap types, and systemic conditions (e.g., long-term smoking, use of vasoactive drugs, body temperature, and mean arterial pressure) could influence TcPCO_2_ measurement results. Although this study excluded patients with severe ventilatory dysfunction (e.g., COPD) from the inclusion criteria, and a review of enrolled patients revealed no individuals with factors known to affect flap blood supply (such as long-term smoking or diabetes), selection bias in patient enrollment remains. Thus, subsequent research should further expand the sample size, enhance stratified analysis, control for confounding factors, and explore differences in TcPCO_2_’s ability to reflect flap blood supply across different tumor types and flap types. In this study, free flaps were continuously monitored preoperatively, intraoperatively, and postoperatively, with measurement times treated as the sample size for analysis. Despite using a mixed-effects model for statistical analysis (with patients set as a random effect to correct for data correlation), the risk of Type I errors may still be elevated. Therefore, subsequent research should further increase the sample size, enhance follow-up monitoring of flaps, and analyze differences between independent samples to better explore the diagnostic efficacy of TcPCO_2_.

TcPCO_2_ monitoring offers the advantage of convenient operation and enables continuous, non-invasive monitoring of free flaps in clinical practice. For populations at high risk of insufficient flap (e.g., patients with diabetes, a history of smoking, or large flap sizes), continuous TcPCO_2_ monitoring can help clinicians more accurately assess flap blood supply status. Meanwhile, clinical observation of intraoral flaps (e.g., flap color, and temperature) is easily affected by oral secretion coverage or tissue swelling obstruction, and traditional monitoring methods (e.g., palpation of vascular pulses and Doppler ultrasound) have limitations in operating on deep flap regions. In contrast, TcPCO_2_ enables dynamic monitoring via a surface probe, effectively addressing the aforementioned shortcomings of traditional methods. Although the cost of a single transcutaneous blood gas analyzer is relatively high, its long-term benefits are significant: from the perspective of “avoiding additional costs from complications,” once insufficient flap occurs, additional medical expenses (e.g., surgical debridement and secondary flap transplantation) are required, and patients’ hospital stays are prolonged, resulting in overall economic losses far exceeding the investment cost of TcPCO_2_ monitoring. However, the widespread promotion of TcPCO_2_ still faces several challenges: First, professional training for staff involved in TcPCO_2_ monitoring is necessary. Second, to advance its further promotion, future efforts should focus on three areas: developing low-cost equipment, simplifying electrode design, and optimizing instrument operation procedures. Furthermore, accumulating more real-world application data is essential to ultimately transform TcPCO_2_ from a “research tool” into a “routine clinical technique.”

## 5. Conclusions

In the monitoring of blood supply after free flap transplantation in patients with oral tumors, TcPCO_2_ exhibits high specificity: when TcPCO_2_ levels increase (≥66 mmHg), it can serve as an effective diagnostic indicator for early identification of insufficient flap, Compared with TcPO_2_, TcPCO_2_ is less affected by external factors and has more stable values; therefore, it holds higher practical value in clinical practice. However, this study still has limitations. In subsequent research, it is necessary to further expand the sample size, include “early reversible perfusion disorders” in the research scope, and simultaneously explore differences in TcPCO_2_’s ability to reflect flap blood supply under different tumor types and different flap types.

## Figures and Tables

**Table 1:** Patient baseline characteristics.

Characteristics	Normal (93)	Insufficient (28)	*p*
Sex/*n* (%) Male Female	69 (74.2) 24 (25.8)	22 (78.6) 6 (21.4)	0.717
Average age (years)	55 ± 13	54 ± 11	0.898
Average weight (kg)	66 ± 14	66 ± 14	0.805
Average height (m)	1.69 ± 0.08	1.68 ± 0.07	0.796
Malignant tumor/*n* (%) Tongue Gingival Mandible The bottom of the mouth Cheek Others	37 (39.8) 23 (24.7) 10 (10.8) 6 (6.5) 9 (9.7) 8 (8.6)	15 (53.6) 5 (17.9) 2 (7.1) 2 (7.1) 3 (10.7) 1 (3.6)	0.868
Free flaps/*n* (%) Anterolateral thigh Forearm Fibular myocutaneous	59 (63.4) 20 (21.5) 14 (15.1)	19 (67.9) 4 (14.3) 5 (17.9)	0.740

**Table 2:** Comparison of TcPO_2_ and TcPCO_2_ levels between normal and insufficient flap groups.

Group	TcPO_2_	TcPCO_2_
Insufficient	3 ± 4	85 ± 29
Normal	59 ± 47	44 ± 17
*p*	<0.001	<0.001

**Table 3:** The diagnostic efficacy of TcPCO_2_ and TcPO_2_ + TcPCO_2_ in determining insufficient flap.

Outcome	TcPO_2_	TcPCO_2_	TcPO_2_ + TcPCO_2_
AUC	0.905	0.912	0.955
Cut-off (mmHg)	16	66	
95%CI	0.853–0.956	0.856–0.968	0.921–0.990
Sensitivity	0.786	0.536	0.821
Specificity	0.860	0.946	0.968
PPV	0.62	0.750	0.885
NPV	0.930	0.81	0.947
Accuracy	0.843	0.851	0.934

Abbreviations: PPV, positive predictive value; NPV, negative predictive value.

## Data Availability

The original contributions presented in this study are included in the article. Further inquiries can be directed to the corresponding author.

## Abbreviations

COPD	chronic obstructive pulmonary disease
PPV	positive predictive value
NPV	negative predictive value
